# Halal Criteria Versus Conventional Slaughter Technology

**DOI:** 10.3390/ani9080530

**Published:** 2019-08-05

**Authors:** Fouad Ali Abdullah Abdullah, Gabriela Borilova, Iva Steinhauserova

**Affiliations:** Department of Meat Hygiene and Technology, Faculty of Veterinary Hygiene and Ecology, University of Veterinary and Pharmaceutical Sciences Brno, Palackeho tr. 1946/1, 612 42 Brno, Czech Republic

**Keywords:** stunning, slaughter, halal criteria, animal welfare

## Abstract

**Simple Summary:**

While legislation can be implemented, amended or revoked according to the requirements of a given time period, this is very difficult or even impossible with religious laws due to their sanctity. Faithful Muslims believe in the sanctity of the main Islamic laws (Quran and Hadiths) because, according to them, they originated from heavenly and divine sources (God and his prophet Muhammad). The technological means of the meat industry are constantly evolving to meet the requirements of the present day and may sometimes be inconsistent with ancient religious norms. Recently, greater attention has been paid to the halal meat industry due to the increased size of the Muslim community in Europe; the opportunity of producing and exporting meat and meat products to the Islamic world; and the ease of delivery, trade and transport. The purpose of this study is to highlight some of the controversial aspects of modern technological means and the principles of the halal meat industry. The controversial aspects that this study deals with include the practices of modern slaughter (animal fasting prior to slaughter, the animal’s body position during slaughter, the location of the incision during bleeding, stunning and mechanical slaughtering). This review may contribute to raising awareness among producers and consumers and to finding means of technology compatible with halal criteria.

**Abstract:**

The halal meat industry is today a reality in many regions of the world, including the European Union. The main religious laws in the area of halal meat production were legislated in ancient times and may be unchangeable due to their sanctity perceived by faithful Muslims, while the modern technology used in the meat industry is constantly evolving and being updated. The objective of this study is to highlight the points of controversy between the principles of halal and the technological means currently used in the meat industry. Modern slaughter practices, including animal fasting prior to slaughter, animal body position, the location of the incision during slaughter, stunning and mechanical slaughter, are reviewed. The purpose of preslaughter feed availability according to halal criteria could be to ensure greater welfare for animals, though feed withdrawal is necessary today. Although there is no clear unified opinion among the Islamic sects, reversible stunning of animals is generally accepted. A neck cut at a higher position than the conventional low cut in cattle may reduce the compromise in welfare (the onset of unconsciousness), minimise false aneurysm and be compatible with halal criteria. This study may contribute towards consideration being given to technology that is not in conflict with the religious legislation, while at the same time meeting the requirements of the modern meat industry.

## 1. Introduction

The global population of Muslims reached to 1.8 billion in 2017, and their population is increasing annually [1]. The markets for halal food are growing at an extraordinary rate in line with the growth in the Muslim population. The amount of money Muslims spent on food and beverage in 2017 was valued at US$1.3 trillion, which represents total halal food market spending [1]. Currently, the halal food market accounts for a share of around 16% of the global food industry and may constitute around 20% of global trade in food production in the future [2]. The food industry in Europe has begun to invest in halal food production. Fresh halal meat has been added to the ranges offered by certain European retailers, and some European manufacturers are exporting their products to the Islamic world. The Netherlands serves as a channel of halal food to the European market, the Middle East and Africa, as it ranks first globally in the field of halal food storage and warehousing [3]. According to the DinarStandard synthesis and analysis report of 2015, Brazil, India, Argentina, Russia and France are the leading halal meat exporters to Organisation of Islamic Cooperation (OIC) countries, representing 28.5% of the total halal meat market. Saudi Arabia, followed by Malaysia, the United Arab Emirates, Indonesia and Egypt are the top five halal meat importers, representing 42% of the global halal meat market [4]. Halal criteria should be observed for all products produced to meet the needs of Islamic consumers. These criteria are associated with the origin, nature and processing methods of food production [5]. Halal food products are defined as food that is produced according to halal tradition [6]. Islamic dietary criteria divide food into permitted (halal) and prohibited (haram) to Muslim consumers. These criteria are mentioned in the Quran and the Hadith (the sayings of the prophet Mohammed) and are explained and interpreted by Islamic scholars [7]. Islamic dietary criteria are binding on Muslims and should be observed at all times [5]. It is, therefore, necessary to perform checks on the halal status of food products provided for Muslim consumers. Basically, according to the Quran (Chapter V, Verse 3), Sunnah (the actions of the prophet Mohammed) and doctrines (scholars), the following kinds of animals are not halal [4]:

- dead animals
- pigs
- all animals slaughtered without the name of Allah being pronounced on them
- animals with long pointed teeth or tusks
- primates, reptiles (except spiny-tailed lizards) and amphibians
- donkeys, mules (horses are not forbidden) and *Lycaonpictus* (African wild dog)
- most insects
- only aquatic animals that are harmful to human health
- blood (from any animal) and products made or sourced from blood.

The Arabic word “Zabiha” refers to the bleeding of animals intended for meat production following the Islamic criteria of Islamic ritual slaughter, also known as halal slaughter [7]. In the Western world, several unregulated halal certification bodies have been created in an attempt to assure Muslim consumers that products with halal certification meet the requirements of Islamic dietary law [8]. The standard certificates should comply with halal requirements to be acceptable for customers. The World Halal Council (WHC) is currently the largest certification organisation and oversees around 41 halal certification agencies from different countries. The OIC, which includes 57 member states, has developed general guidelines on halal food for its members worldwide [2]. Although these halal certification bodies are unregulated and often operate according to different halal standards, they are considered enforcers of halal dietary laws by many Muslims, especially in minority Muslim population countries where there is a possible risk of cross contamination with non-halal raw materials (such as pork) [9]. Recently, the halal meat industry has been the focus of research of many authors from different viewpoints, examining the principles of halal meat [4], halal control points (HCPs) in meat processing [5,7], the aggregate of Islamic dietary and Hazard analysis and critical control points (HACCP) principles [10] and religious versus legal understandings of halal slaughter [11]. New technological practices and processes in the meat industry are constantly being developed in order to be more economical and hygienic, to comply with increasing consumer demand and, at the same time, to be in compliance with animal welfare [11]. Nevertheless, these technological means may conflict with halal criteria in some respects. The aim of this review is to highlight the compatibility of modern technological procedures with halal principles regarding slaughter practices.

## 2. Fasting of Animals Prior to Slaughter

It is recorded that the Prophet Muhammad said, “When one of you slaughters, let him complete it”, meaning that one should sharpen the knife well and feed, water and soothe the animal before killing it (as mentioned in Sahih Muslim, Book 21, Chapter 11, Number 4810) [12]. The principles of Sharia (Islamic) law therefore prefer the animals to be well fed by providing free access to feed and drinking water while waiting prior to slaughter [13]. In the present day, the fasting of ruminants during periods of transportation and lairage at slaughterhouses is recommended to reduce the volume of the gut contents and, therefore, the quantity of bacteria, thereby reducing the risk of carcass contamination during dressing [14]. However, maximum periods of fasting/feeding and journey times for different animal species are given in Council Regulation (EC) No. 1/2005 [15]. Council Regulation (EC) No. 1099/2009 states, “Animals which have not been slaughtered within 12 h of their arrival shall be fed, and subsequently given moderate amounts of food at appropriate intervals” [16]. This means that the feed withdrawal period preslaughter is up to 12 h. The issue of preslaughter feeding and fasting is closely associated with the duration of transport. Energy and fluid balance must be taken into account when animals are prepared for transportation [17]. Heat stress is an important factor related to fasting and pre-transport feeding. In cattle, the rumen is the greatest source of heat production due to exothermic fermentation processes through feed digestion, which take about 4–6 h from feeding [18]. It is, therefore, recommended to fast animals and then transport them to the slaughterhouse particularly in hot climate areas, and this may also prevent transport sickness.

Currently, there is agreement about the necessity of preslaughter fasting of animals in the European Union [15]. In the rest of the world, the number of hours of preslaughter fasting for animal species is variable according to the legislation of the individual countries. A preslaughter fasting period of 12 h is stipulated as the minimum time for cattle and as a maximum for broilers by the regulations of the Brazilian Ministry of Agriculture [19,20]. Prolonged feed deprivation has a negative influence on certain characteristics of meat quality and increases physiological stress parameters in lambs; for this reason, fasting for more than 24 h is not recommended [21]. O’Neill et al. [22] found that meat tenderness was not affected negatively when feed was available to cattle up to 3 h preslaughter. However, providing feed for animals preslaughter was intended to ensure animal welfare by Islamic (Sharia) criteria, while feed withdrawal is necessary today. It is important to explain to Islamic jurists that the preslaughter fasting of animals for a restricted period (a few hours, according to the animal species) is not related to animal welfare but has great technological advantages [15,16,17], which could convince them to issue a fatwa (Islamic nonbinding legal opinion) to permit it.

## 3. Stunning

Stunning is one of the most controversial technological operations in the halal meat industry. Stunning is not mentioned in the Quran or the Hadith literature. Stunning raises concerns regarding the fulfilment of the requirements and principles of halal slaughtering that are mentioned in the primary sources of Islam. Such concerns relate to the fact that the animal is not alive at the time of slaughter and is only partially exsanguinated due to stunning [11]. According to Council Regulation (EC) No. 1099/2009, the stunning of animals prior to slaughter is a statutory requirement in Europe and applied in order to render animals unconscious [16]. The definition of “stunning” provided by Council Regulation (EC) No. 1099/2009 means any intentionally induced process which causes loss of consciousness and sensibility without pain, including any process resulting in instantaneous death [16]. The objective of stunning is to prevent anxiety, pain, suffering and distress in animals before and during the slaughter process [23]. Many complications result from slaughtering cattle and small ruminants without stunning, including pain and stress related to restrained movement [23], pain resulting from the incision and stimulation of nociceptors in the wound [24,25], delay in the time to loss of consciousness and related distress [26] and blood aspiration into the respiratory tract and associated distress [27]. False aneurysms can develop in halal-slaughtered cattle (without stunning) due to delays in the loss of consciousness leading to obstruction of the flow of blood [28]. Council Regulation (EC) No. 1099/2009 indicates, “Slaughter without stunning requires an accurate cut of the throat with a sharp knife to minimise suffering”. In addition, animals that are not mechanically restrained after the cut are likely to endure a slower bleeding process and, thereby, prolonged unnecessary suffering. Animals of bovine, ovine and caprine species are the most common species slaughtered under this procedure. Therefore, ruminants slaughtered without stunning should be individually and mechanically restrained. Beside the religious reason, stunning is often not practiced worldwide due to other reasons, such as lack of available stunning tools and equipment, cost of the equipment, lack of technical training on the usage of stunning tools and resources [29]. Slaughter of animals without stunning requires a highly skilled staff to handle the animals and perform the bleeding. The Canadian Food Inspection Agency [30] demands that employees who work with animals must be trained in

- monitoring of the indicators for loss of consciousness
- the ability to detect (in compliance with established performance criteria in animal welfare) that loss of consciousness was not achieved during bleeding
- implementation of corrective actions if any deviations occur in order to prevent the suffering of the individual animal (e.g., postcut stunning)
- understanding that introducing multiple or additional cuts or sawing actions (nonfluid motions) is not allowed as a corrective action
- making sure that the animal is unconscious (by monitoring multiple indicators) prior to hanging it up
- assessment of animals after bleeding to ensure that the animal is dead prior to further procedures, such as dressing or manipulation of the incision area for religious purposes.

If the staff performing bleeding is poorly trained or incompetent, the interval between restraint and cutting can unacceptably increase suffering and negatively influence animal welfare [23].

Although European legislation (Council Regulation No. 1099/2009) permits religious slaughter, which is usually conducted without stunning, several countries (e.g., Sweden, Denmark, Norway and Switzerland) have already prohibited the slaughter of animals without prior stunning [31]. In fact, all advocates of preslaughter stunning, as well as advocates of religious slaughter methods, insist that stunning is the most humane method [32].

There are differences among Muslims regarding the acceptability of preslaughter stunning practices. While it is accepted in certain countries such as Malaysia (that follow the Shāfʿī school of thought), it is not accepted in Pakistan (which follows the Ḥanafī school of thought) [33]. Currently, the attitude of Muslims to stunning before slaughter can be divided into three main opinions: acceptance with specific requirements (reversible stunning); rejection due to the incompatibility with religious rules and insufficient bleeding (as blood is not considered halal material); and not sure yet or require certain assurances [32]. A survey carried out in England by EBLEX [34] found that 76% of Muslims (out of 1000 respondents) refused the stunning of animals before slaughter and preferred to buy meat from suppliers selling only meat from nonstunned animals. Despite its limited representativeness, this study shows that even Muslims living in Western Europe prefer to consume meat from animals that have been slaughtered by traditional methods and refuse meat from animals rendered unconscious before killing. Although some Muslim authorities have issued a favourable opinion on some stunning methods, so far, there is no general approval of stunning methods used for cattle in the European Union, which may cause economic losses for producers of halal beef, as consumers may turn to other foreign sources [8]. Australian national standards require the stunning of animals prior to slaughter for welfare reasons. However, halal slaughter using reversible stunning is permitted [35]. A small number of slaughterhouses in Australia have even obtained permission from the local authority to perform halal slaughter without the stunning of animals (only for cattle and sheep) but under the condition of immediate stunning after cutting the throat (only for cattle). This means that sheep are slaughtered without stunning, even after throat incision, except in cases of distress (e.g., vocalising, butting or attempting to flee) or when loss of consciousness takes too long, requiring immediate stunning after the neck incision [36,37]. Stunning of animals prior to slaughter is usual and logical due to animal welfare. If it is conducted immediately after throat cutting, it could be performed in order both to reduce animal suffering until loss of consciousness and to meet halal requirements, being therefore humane and religiously correct at the same time.

Farouk et al. [38] noted that reversible preslaughter stunning technology is constantly evolving in the meat industry sector in order to improve animal welfare during slaughter as well as meet the requirements of halal slaughter. The authors confirmed that new systems that are still in development could be applied in the halal meat industry, where they will be able to fulfil the requirements of both halal slaughter and the legislation. For example, single-pulse ultrahigh current (SPUC) [39] and microwave energy [40,41], which are in experimental trials, could be further developed to be used as commercial stunning systems that may meet the requirements of halal slaughter. Some of these technological developments have been well summarised in previous reviews [42,43]. According to Fuseini et al. [44], a survey study in the United Kingdom reported that most Islamic scholars and consumers agreed that reversible stunning complies with the principles of halal. The Department of Islamic Development Malaysia [45] has provided specific guidelines for reversible halal preslaughter stunning. For example, in the case of broilers with a body weight of 2.40–2.70 kg, the use of 2.50–10.50 V for 3.00–5.00 s is recommended. In this regard, the US mode of immobilisation (electrical stunning, ES) of poultry is more suitable for halal requirements than the European one. Low-voltage ES (10–25 V) and high-frequency (500 Hz) systems are used for bird immobilisation in the United States. Such a low voltage means that the birds may regain consciousness within 2 min after stunning if not bled. In the European Union, irreversible stunning (120–150 mA per bird; 50–400 Hz) is used and represents fatal stunning, thus not meeting halal meat criteria [16,46,47]. Although the voltage of electrical stunning and the size of the birds have been standardised, some birds die due to the stunning process prior to slaughter or a delay in the time of the slaughter process, leading to poor blood drainage [33,48]. The ability of poultry to withstand the same voltage current and remain alive after electrical stunning differs even within a particular weight parameter. This is the reason for the unacceptability of stunning with an electric current in the halal poultry industry [48]. Potentially painful pre-stun shocks can occur during water bath stunning if a bird’s wing or any other part of the bird makes contact with the live water before the head [49]. European legislation states that electrical shocks before stunning must be prevented [16], which can be ensured by properly designing the water bath to include an entry ramp. Mis-stuns can occur particularly when the flock is unequal in size and smaller birds arch their neck or lift up due to a pre-stun shock and fly the full length of the water bath stunner [49]. The advantages of properly applied high-frequency preslaughter electrical stunning in ruminants and poultry were reported by Sabow et al. [50] and include efficient bleeding and improved meat and carcass quality; as it is reversible, it also complies with the minimum requirement of halal slaughter. Nonpenetrative percussive reversible stunning (if it does not lead to neuropathological changes) could be an acceptable method for halal slaughter in some Muslim countries. However, a relatively large incidence of subarachnoid haemorrhage was observed in cattle, which makes this method questionable for the halal meat industry [51]. Gibson et al. [52] found that the ratio of mis-stuns in adult cattle (bulls) when using a nonpenetrating captive bolt stunner was 18%, which is a serious welfare problem. European legislation allows use of this method only for poultry, lagomorphs and ruminants up to 10 kg of live weight in order to ensure that the blow is sufficient to render the animal unconscious and avoid mis-stuns. Fracture of the skull must be avoided [16]. Chandia and Soon [11] confirmed that stunning prior to slaughter can be adopted if it fulfils the three following prerequisites:

- The stunning must be reversible; it must not kill the animal or cause permanent injury.
- Stunning should be performed by a trained Muslim slaughter-man or supervisor under regular monitoring by a competent Islamic authority or halal certification body.
- Halal slaughter animals should never be stunned with stunning equipment used on pigs.

## 4. The Position of the Animal’s Body During Slaughter

**Figure 1:**
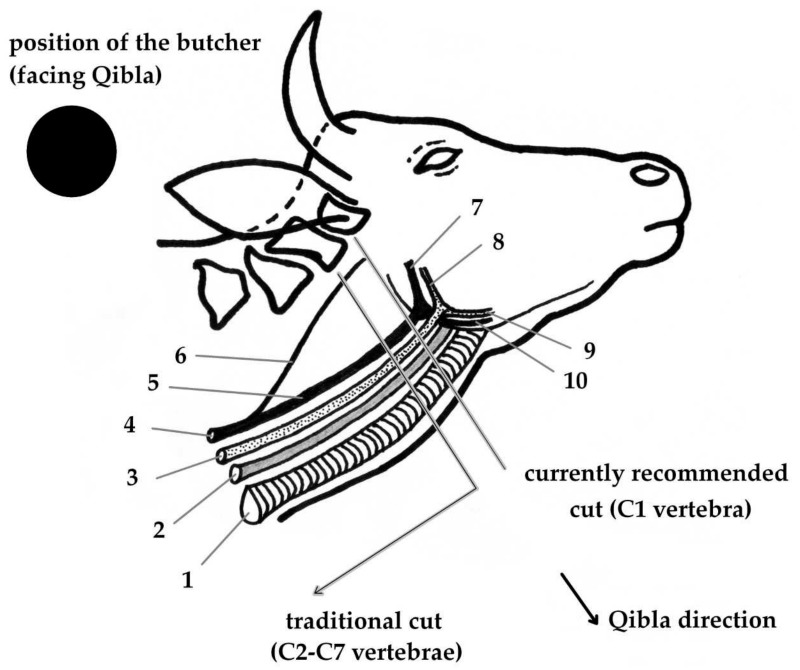
Location of incision during the halal slaughter of cattle (ruminants). 1: *trachea*; 2: *oesophagus*; 3: *vena jugularis externa dextra*; 4: *arteria carotis communis*; 5: *arteria carotis externa dextra*; 6: *arteria carotis interna dextra*; 7: *arteria maxillaris*; 8: *vena maxillaris*; 9: *vena linguofacialis*; 10: *truncus linguofacialis*.

The appropriate restraint of animals prior to slaughter is extremely important in order to conduct the slaughtering process properly, especially for halal slaughter without stunning. The accuracy of the incision could be negatively affected during halal slaughter if animals panic or are agitated by the restraint [53]. The use of less-stressing restraint techniques leads to improved animal welfare and slaughter operative safety, as well as the improved quality of the product [54]. The position of animals during halal slaughtering should be that they are laid on their left flank and preferably facing the Qibla (in the direction of Makkah in Saudi Arabia). This means that at the onset of the performance of the incision, the slaughterer should face towards the Qibla and the animal’s neck (location of incision) should be turned to face the Qibla (Figure 1). During slaughter, according to the Islamic rules of slaughter as indicated by the Hadiths, animals should be shackled and elevated only after bleeding and when the animal has lost consciousness [12]. These are the historical practices of animal slaughtering in Islam. The purpose of this slaughter position (lying on the left flank) is, according to its supporters, to increase the probability of maximum blood drainage by the pressure of the body on the heart [13], as consumption of blood is forbidden for Muslims (Quran 2:173, 5:3, 6:145, 16:115). The idea of greater blood loss from sheep slaughtered in the horizontal position than from those hung vertically is supported by some studies [55,56]. Several methods of restraint (particularly for ruminants) have been used in modern halal slaughterhouses over the years, such as the hoisting of conscious cattle by the hind leg, restraining in the upright or standing position, restraining cattle by inverting them on their backs and lateral recumbency [8,57]. The hoisting of cattle by the hind leg when fully conscious, which is still used in some countries such as Turkey, causes significant pain and unnecessary suffering for animals resulting from their heavy weight and the anatomy of the digestive system [58,59]. Such inhumane animal restraint is against the teaching of Islam, EU legislation and animal welfare. Upright restraint performed in pens known as American Society for the Prevention of Cruelty to Animals (ASPCA) boxes is designed with a chin lift that stretches the neck to provide easy accesses for halal incision performance [8]. The disadvantages of the upright restraint method during the halal slaughter (without stunning) of cattle are the possibility of blood aspiration into the lungs, poor bleeding (clamping of the blood vessels of the neck against the head restraint) and delayed loss of consciousness. The upright restraint method is currently widely used by European halal authorities, although it is not preferred by certain groups of Muslims [27]. Dialrel [59] reported that the restraint of cattle on their backs provides poor animal welfare, confirming postmortem contamination of the trachea and larynx with blood and gut content depending on the position and depth of the ritual incision. The restraining of cattle in the lateral recumbency position (at a 90° angle, lying on their sides) is the most preferable restraint method for most halal slaughterhouses because it is the most suitable method regarding compliance with halal criteria [8]. Although the cattle do not suffer from problems associated with pressure on the diaphragm, aorta or major veins if the lateral recumbency restraint method is used, pressure on other internal organs may occur [59,60]. Restraint in a V-shaped or straddled conveyor with full or half inversion in a rotary pen and an upright restraint system are useable methods in red-meat animal slaughterhouses [57]. The common position of poultry at slaughter is vertical hanging on shackles [61]. Velarde et al. [23] assessed and compared different restraint systems for animals during halal slaughter, including turning the animals by 45°, turning them on their side (i.e., by 90°), turning them on their back (a 180° turn) and restraining animals in an upright position. The study found that struggling and vocalisation were observed in cattle with all restraint systems, though the highest levels were reported when the cattle were turned on their sides. The study also found that the percentage of adequately bled animals was highest when animals were turned on their sides, while it was lowest when slaughtering processes were conducted with the animals in an upright position. According to the same research, the sufficient bleeding of animals turned on their sides at slaughter was attributed to the fact that the time to loss of posture (recumbent and hypotonic animals) is shortest in such a position. Significant variation in the number of cuts was reported between restraint systems, with more cuts performed on cattle restrained in the upright position (nine cuts) in comparison with inversion at 180° (five cuts) and 90° (three cuts). This study indicated that the interval between restraining and cutting the neck in sheep slaughtered without stunning was lowest in animals manually restrained on their side [23]. Warriss and Leach [62] indicated that the slaughter position affects bleeding. Khalid et al. [63] found that blood loss after the slaughter of lambs increased significantly when carcasses were hung vertically rather than kept upright in a V-restrainer. This greater bleeding of animals is most likely due to the change in animal orientation and gravity [64]. More effective bleeding in animals hung vertically by shackling the hind legs in comparison with animals bled in a horizontal position was also reported in cattle [65]. The possibility of blood aspiration in the respiratory tract during the slaughter of cattle has been reported. Blood aspiration in the respiratory tract during the slaughter of cattle was shown in the upright (standing) position by Gregory et al. [27] and the inverted position by the European Food Safety Authority [46]. It is known that the method of halal slaughter was conducted manually in the past by people without the help of machinery, for which reason the position of the animals during halal slaughter (lying on the left flank) and facing the Qibla could be suitable in such a case. However, it is difficult to fulfil the conditions for the animal’s position during halal slaughter in the case of poultry, when the birds are restrained and hanging vertically and mechanical slaughter plays a major role. Although the Islamic law states “all the animals except camel” [13], according to a fatwa, lying on the left flank is preferable but not mandatory for halal slaughter of poultry.

## 5. The Location of Cutting (Incision) During Slaughter

Hadith states that “the cut must be made on the neck, just below the gullet and the core of the neck”. The Hadith moreover mentions that “the jugular veins” and “the carotid arteries must be cut, in addition to the oesophagus and the trachea” [66]. The slaughter act must be performed by prompt incision with one uniform continuous movement and without any interruption, uncertainty or unnecessary delay. Furthermore, the incision must be performed at the ventral aspect of the neck near the lower jaw and must not reach the spine. This means that the head should not be completely separated from the body during slaughter [67], which can delay loss of consciousness due to blood continuing to flow to the brain through the vertebral arteries [28,68]. The preamble of Council Regulation (EC) No. 1099/2009 indicates that slaughtering bovine, ovine and caprine species without stunning requires accurate cutting of the throat using a sharp knife to minimise suffering, adding that animals under this procedure that are not restrained mechanically after the incision are likely to endure a slower bleeding process and consequently prolonged unnecessary suffering [16]. Minimally painful and complete bleeding is required during halal slaughter, which is difficult to perform in large animals [69]. Previous researchers have indicated an association between the location of the cut and the onset of unconsciousness during slaughter without stunning, such as in halal slaughter. It has been found that cutting the neck at a position parallel to the first cervical vertebra (C1) almost eliminates the development of false aneurysm compared with the conventional C2+, thereby minimising the risk of restricted exsanguination [28,70]. Gibson et al. [70] found that the performance of the neck cut at a higher position than the conventional low cut on the neck reduced the time to loss of posture (onset of unconsciousness) in halal-slaughtered cattle, thereby minimising subsequent distress such as false aneurysm. The development of false aneurysm occurs due to the contraction of the severed carotid artery within its surrounding connective tissue sheath, which can subsequently block the flow of blood from the artery [26,68]. The recommended position of incision is depicted in Figure 1. Gibson et al. [70] suggested that adoption of a high neck-cut tactic in halal slaughter (without stunning) may reduce the compromise in welfare associated with the delayed time to loss of consciousness. It may be appropriate to find and apply such means in the meat industry that take into consideration both animal welfare and religious aspects.

## 6. Mechanical Slaughter (Poultry)

In general, and according to Islamic principles, only Muslims or “People of the Book” (Christians and Jews) that are familiar with the Islamic procedure of animal slaughtering (ritual slaughter) can perform it.

A sharp knife free of scratches and nicks of a suitable length (two to four times the size of the neck of the animal) should be used to perform slaughter properly [71,72]. There are no special types of knifes determined for halal slaughtering as there are for the kosher slaughtering of animals [4]. During slaughtering itself, the specific phrase in the Arabic language “Bismillah, Allahu Akbar”, which means “In the name of Allah, Allah is the greatest”, should be pronounced by the slaughter-man aloud, and the animal should be alive. This is in accordance with commands given in the Quran: “So eat of that over which the Name of God was pronounced” (al-An^,^ am, 118). The slaughter-man repeating this pronouncement aloud when slaughtering each bird in large-scale poultry processing plants, where 4000–12,000 birds are processed each hour on a single line, is extremely difficult in practice [47,73]. For this purpose, the Standing Committee for Scientific Research and Issuing Fatwas (Islamic rulings) issued a fatwa to the effect that it suffices to pronounce this phrase once when running a mechanical slaughter machine in poultry slaughterhouses [74]. It has been reported that at some slaughterhouses, a recording of the phrase is played during machine slaughter instead of being pronounced, but this does not adhere to the fundamental principles of halal slaughtering [75]. It is thought that the mechanical slaughters performed in halal abattoirs do not adhere to Islamic principles [76,77]. This belief is also confirmed on the basis of the evaluation of the DIALRE project [59] for halal slaughter practices for poultry in European abattoirs. Many practices which could be in conflict with halal slaughtering rules have been recorded in such abattoirs. An automatic horizontal rotary knife for neck cutting performed the procedure in three out of the five poultry abattoirs that practiced halal slaughter with stunning. This slaughtering procedure contradicted halal conditions stating that hand slaughter and performance of the act by the slaughter-man (preferably a Muslim, though the person may be a Christian or Jew), accompanied by the invocation of the blessing before incision, is required [23]. Besides, improper neck cutting of chickens by a rotating blade is possible, which negatively influences blood loss and animal welfare. In general, continuous monitoring of such mechanical practices in all poultry slaughterhouses by person or the use of a closed-circuit television is necessary to ensure the correct neck position of birds [75].

Currently, halal slaughter (hand slaughter without stunning) practices are unable to compete with the standard operating procedures of high-speed, high-throughput abattoirs in meeting the enormous demand for meat [11]. Halal slaughter, which requires workers instead of machines, has economic consequences that increase the price of the meat, which means that consumers pay a premium for halal meat.

## 7. Conclusions

The trend of all-encompassing modernity does not exclude any area of life. Practices that were adopted in the past are not used today and what we consider satisfactory today may not be suitable in the future. The main religious laws and regulations in the halal meat industry were promulgated more than 1400 years ago. There is a segment of Muslims who believe that religious laws are valid for all times and places and call for their application today without any change or modification because they believe that these principles are heavenly. The sources of the provisions and legislations concerning halal meat are the Quran, Sunnah and Islamic scholars. Ijmā (consensus of legal opinion) and Qiyās (reasoning by analogy) are also sources of jurisprudence which play an important role in the interpretation and application of slaughter practices, such as acceptability of several stunning methods. Ijmā and Qiyās have secondary importance in comparison with the main and very important sources of Islamic legislation, including the Quran and the Sunnah. Furthermore, these two sources of Islamic legislation do not have the same importance and effectiveness in all of the Islamic legal schools (Madhhab), where the Sunni interpretations are different than Shi’a interpretations. For this reason, there are differences among Muslim countries about the acceptability of stunning methods. The strict adherence of Muslims to these religious instructions depends on the importance of the source of their legislation, including the Quran and Sunnah or Islamic scholars. Our purpose is not to praise or condemn the laws of the halal meat industry or to demonstrate the efficiency of modern meat technology but to highlight the intersections and the areas of consonance and dissonance between the two aspects. The meat industry can take these observations into consideration and provide technology that is appropriate for religious laws. Suitable modern technological means and practices in accordance with halal criteria in the meat industry are the reversible stunning of animals, restraint of animals in a lateral recumbency position (for ruminants) and performance of neck incision at a position higher than the conventional low incision in cattle in order to reduce the possibility of the occurrence of false aneurysm. Hand-cutting (manual slaughter of poultry) nonstunned slaughter, which is more dependent on employees than technological means, is more expensive and time consuming; for this reason, it faces serious difficulties in competing with current slaughtering practices. This research does not deal with the technological processes that are conducted after slaughter, such as skinning, evisceration, splitting, washing and dressing of carcasses, meat cutting and utilisation of meat cuts, due to a lack of religious (Islamic) texts in this regard and finds no conflict with the usual technological practices. The application of all the principles of halal is extremely difficult and complex in practice due to the lack of a unified vision between various sects of Islam. Globalisation and new technologies have both negative and positive effects on the practical application of the rules and laws of halal meat production. It is necessary to create technology that is not in conflict with religious principles while keeping pace with modernisation at the same time.
